# Marine Collagen: A Promising Biomaterial for Wound Healing, Skin Anti-Aging, and Bone Regeneration

**DOI:** 10.3390/md20010061

**Published:** 2022-01-10

**Authors:** Sarah Geahchan, Parnian Baharlouei, Azizur Rahman

**Affiliations:** 1 Centre for Climate Change Research, University of Toronto, ONRamp, Toronto, ON M5G 1L5, Canada; sarah.geahchan@mail.utoronto.ca (S.G.); parnian.baharlouei@mail.utoronto.ca (P.B.); 2 Department of Pharmacology and Toxicology, University of Toronto, Toronto, ON M5S 2E8, Canada; 3 Physiology and Human Biology, University of Toronto, Toronto, ON M5S 1A8, Canada; 4 A.R. Environmental Solutions Inc., ICUBE-University of Toronto, Mississauga, ON L5L 1C6, Canada

**Keywords:** marine collagen, wound healing, bone regeneration, collagen peptides, antiaging, osteoporosis, osteoarthritis, fish collagen, marine sponge

## Abstract

Marine organisms harbor numerous bioactive substances that can be utilized in the pharmaceutical and cosmetic industries. Scientific research on various applications of collagen extracted from these organisms has become increasingly prevalent. Marine collagen can be used as a biomaterial because it is water soluble, metabolically compatible, and highly accessible. Upon review of the literature, it is evident that marine collagen is a versatile compound capable of healing skin injuries of varying severity, as well as delaying the natural human aging process. From in vitro to in vivo experiments, collagen has demonstrated its ability to invoke keratinocyte and fibroblast migration as well as vascularization of the skin. Additionally, marine collagen and derivatives have proven beneficial and useful for both osteoporosis and osteoarthritis prevention and treatment. Other bone-related diseases may also be targeted by collagen, as it is capable of increasing bone mineral density, mineral deposition, and importantly, osteoblast maturation and proliferation. In this review, we demonstrate the advantages of marine collagen over land animal sources and the biomedical applications of marine collagen related to bone and skin damage. Finally, some limitations of marine collagen are briefly discussed.

## 1. Introduction

The extracellular matrix (ECM) plays important roles in the physical integrity of cells, where it is involved in cell proliferation, differentiation, migration, and adhesion [1,2,3,4,5,6]. Collagen is the main structural protein in the ECM and connective tissue of animals. In mammals, collagen protein is highly abundant and mainly localized in the ECM of fibrous connective tissues, such as the tendon and skin [7,8,9,10]. It plays key structural roles by supporting the formation, tensile strength, and flexibility of joints [11,12,13,14,15]. Collagen types I, II, III, V, and XI are able to form fibrils that are necessary for structural support and resistance to mechanical stress in connective tissues [16,17]. Type I collagen is the most abundant form and is mainly present in the tendons and skin [18,19,20].

**Figure 1:**
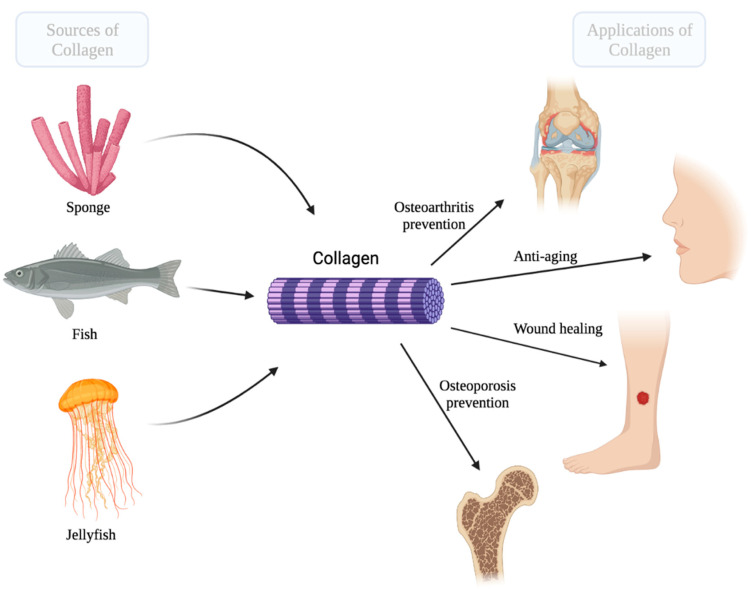
The marine sources of collagen and its main biomedical applications that are discussed in this paper. (Created with BioRender.com accessed on 7 December 2021).

Collagen has numerous biomedical applications ranging from wound healing, bone and tissue regeneration, and drug delivery (Figure 1) [21,22]. Its accessibility, flexibility, and biocompatibility make it a useful biomaterial in several fields [22,23,24]. Collagen is a trimeric molecule made up of three polypeptide alpha-chains, forming highly organized three-dimensional structures capable of resisting mechanical stress and supporting the growth of cells [25,26].

Marine organisms such as fish, jellyfish, sponges, and other invertebrates harbor a significant source of collagen and are highly advantageous over other sources, as they are metabolically compatible, lack religious constraints and are free of animal pathogens [27,28,29,30]. In fact, fish skins are commonly used for type I collagen extraction, as they are not only immensely abundant but also do not have religious restrictions and are not a risk of disease transmission [31,32,33]. Land animals possess many transmittable diseases, which makes them less favorable for use in industries. For example, cattle, although a large source of collagen, pose risks for bovine spongiform encephalopathy (BSE) as well as transmissible spongiform encephalopathy (TSE) [29,34,35]. These progressive neurological disorders affect cattle and can result in life-threatening infections in humans [29]. In addition, some religious constraints on the use of bovines for the pharmaceutical and cosmetic industries are up for debate [35]. These factors make marine sources of collagen a much safer, easier, and promising alternative.

Skin wounds may take a long time to heal and often do not heal completely. Marine collagen isolated from organisms like fish, jellyfish, and sponges has been implicated in several studies on its potential for increasing wound healing rates [36,37,38,39,40,41]. The processes involve increased fibroblast and keratinocyte migration as well as vascularization and epidermal growth [42,43,44]. In addition to accelerating wound healing, marine collagen has also been shown to have anti-aging properties by slowing the aging process in mice [45,46,47,48]. Studies on humans have also shown that marine collagen can reduce wrinkles, improve skin elasticity, and enhance the overall structure and appearance of skin. Furthermore, collagen’s ability to regenerate bone has been shown to be successful in rat models of menopausal osteoporosis [49]. Marine collagen is able to increase bone mineral density and osteoblastic activity, serving protective effects against bone degeneration [49,50,51,52,53]. Collagen has also been shown to induce chondrogenic differentiation and prevent the development of osteoarthritis (OA) [54,55].

Here, we review the potential application of marine collagen in facilitating wound healing and highlight that marine collagen can enhance skin elasticity and thus, reduce the aging process of the skin. Furthermore, we describe the suitability of marine collagen for bone tissue engineering and cartilage formation due to its high biocompatibility. Although some limitations associated with the use of marine collagen exist, it is evident that the advantageous and efficacious potential of marine collagen significantly outweighs its drawbacks.

## 2. Collagen Application in Wound Healing and Anti-Aging

Our skin epidermis is the most important innate defense barrier against all pathogens and plays a significant role in tissue homeostasis [56,57,58]. Skin injuries are difficult to treat yet are becoming increasingly common as a result of burns, infections, scarring, genetic disorders, and other diseases [59,60]. Treatments aim to restore the integrity of the tissue, involving processes such as inflammation, cell division, differentiation, and vascularization. Endothelial permeability enables cell adhesion, which is followed by cell differentiation and maturation [61,62]. Marine collagen has been shown to be an effective biomaterial for wound healing. Collagen can be utilized in various formulations, such as the use of collagen peptides and hydroxylates, or collagen fibers, and scaffold-like structures [44,63].

**Figure 2:**
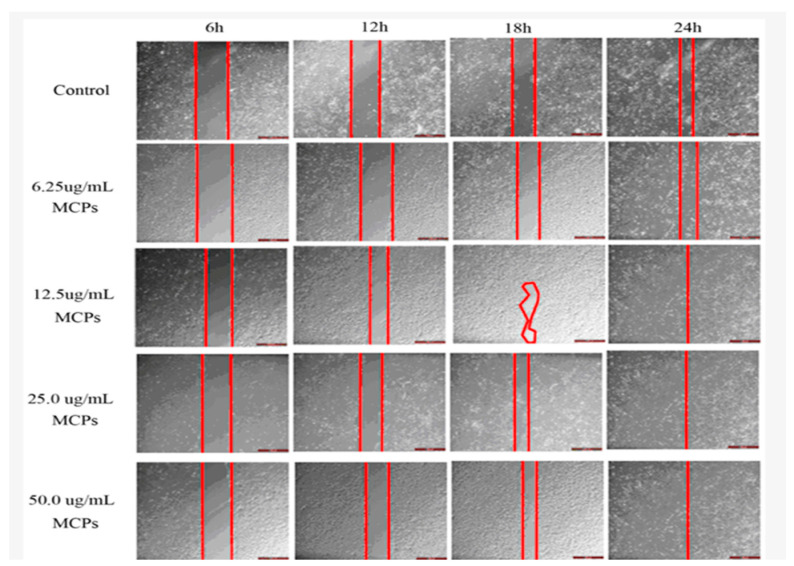
The effect of varying concentrations (6.25, 12.5, 25.0, 50.0 μg mL^−1^) of marine collagen peptides (MCPs) on scratch closure was determined by an in vitro assay [63].

Marine collagen peptides are produced from collagen through both chemical and enzymatic hydrolysis, and their smaller molecular weight increases their water solubility, making them more absorbable [63,64]. Hu et al. used an in vitro scratch assay to demonstrate that marine collagen peptides improve wound closure at concentrations of 50 μg mL^−1^ starting at 12 h post-treatment with collagen (Figure 2) [63]. It was shown that at 50-μg mL^−1^, the cell migration induced was similar to migration seen by 10.0 ng mL^−1^ of epidermal growth factor, a factor that is known to play a crucial role in wound healing. Furthermore, using marine collagen peptides isolated from the skin of tilapia, wounded rabbits treated with collagen healed significantly faster compared to the control group after 11 days [63].

Moreover, Yang et al. isolated collagen peptides from Alaska pollock and demonstrated that oral administration of collagen peptides to wounded rats significantly increased recovery rates compared to the control groups [65]. Hydroxyproline, which promotes the deposition of collagen and thus healing, was shown to be greater in the collagen-treated group (10.6 µg mg^−1^) over time than in the control group (9.25 µg mg^−1^) [65]. On day 12 of healing, the treated groups displayed complete re-epithelialization, and the presence of hair follicles was observed, whereas the control group had poor keratinocyte migration and no hair follicles [65].

**Figure 3:**
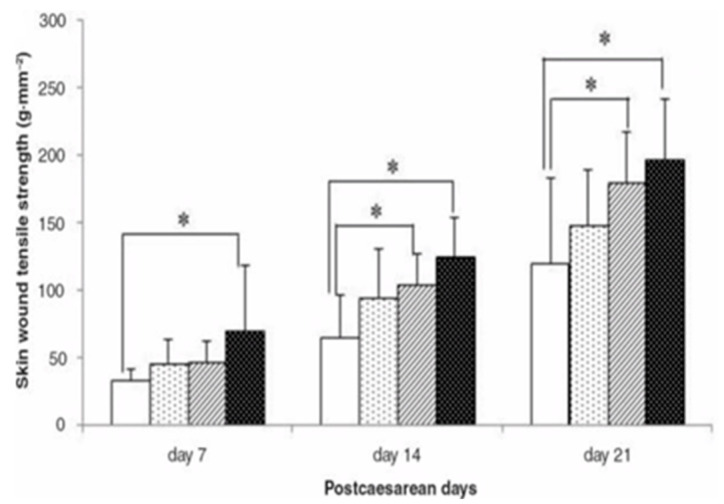
Depiction of skin wound tensile strength at days 7, 14, and 21 in the vehicle- and MCP-treated groups. The vehicle is shown by the white bar, and the MCP-treated groups are represented by the other three bars at increasing doses of 0.125, 0.375, and 1.125 g/kg bw. Data are shown as the mean +/− SD. * significant difference at *p* < 0.05 [42].

Similar to the studies mentioned above, Wang et al. found that marine collagen peptides (MCPs) isolated from salmon skin significantly improved skin wound tensile strength in rats [42]. As seen in Figure 3, the observed improvement was dependent on the dose of collagen peptides and the time after cesarean section [42]. Furthermore, hydroxyproline levels were remarkably increased in the collagen-treated groups compared with the control groups, increasing in a time- and dose-dependent manner [42]. Not surprisingly, rats in the collagen-treated group showed elevated fibroblast proliferation and vascularization at 7 days post-treatment [42]. These results are similar to the experiment of Zhang et al., who demonstrated that marine collagen peptides enhanced wound closure rates in rats, tensile strength at the incision site and collagen deposition [43]. Histological analysis revealed improved vascularization, epithelization, and fibroblast infiltration in the collagen-treated groups [43]. Collagen deposition is an important part of the recovery from skin injuries and the development of granulation tissue, and hydroxyproline, which promotes collagen deposition, was elevated in the treated groups at 7- and 11-days post-injury [43].

Additionally, Pozzolini et al. isolated and purified marine collagen hydroxylates from the marine sponge *Chondrosia reniformis* [66]. Using an in vitro scratch assay, 50 ug/mL of collagen peptide fractions were added, and cells were analyzed at 0, 6, 24, and 30 h post-administration. Compared to the control groups, the treated cells demonstrated fibroblast and keratinocyte migration and proliferation, increasing wound gap closure of both dermal and epidermal cells. Cells were first observed migrating and colonizing the scratch gap area, followed by increased cell proliferation by the 24-h time point [66]. These results suggest the promising wound-healing abilities of the marine collagen hydroxylates isolated from C. *reniformis.* Similarly, hydrolyzed peptide collagen isolated and purified from the jellyfish *Rhopilema esculentum* demonstrated wound healing activity both in vitro and in vivo. First, using a scratch wound-healing assay the authors demonstrated that collagen peptide treated groups had increased cell migration and wound closure dose-dependently at 18-, 36- and 48-h post-treatment [67]. When investigated on wounded mice, collagen peptides (0.9 g/kg) improved wound healing effects, in part by promoting increased expression of chemotactic factors (i.e., β-FGF and TGF-β_1_). These factors play a role in protecting the wound from infection by recruiting inflammatory cells, which can also regulate the migration of fibroblasts and keratinocytes; thus, promoting wound healing [67].

Moreover, Veeruraj et al. isolated astaxanthin, an antioxidant and anti-inflammatory compound as well as acid and pepsin soluble collagen from the squid, *Doryteuthis singhalensis* [68]. Wounded rats were treated with an astaxanthin and collagen combination, and a faster wound healing rate was observed compared to the saline- treated wounds. The study also found that collagen-treated groups had increased epithelization, angiogenesis, keratinization, and presence of collagen fibers, which together contribute to wound healing [68].

Likewise, Chen et al. isolated collagen from marine tilapia skin and bovine skin collagen nanofibers and showed that collagen-treated rat groups had faster wound recovery rates than controls [44]. In addition, the study found that hydroxyproline, a component of collagen, played an important role in the rate of wound healing by promoting re-epithelization. The collagen-treated groups had more fibroblasts, more vascularization, less inflammation, and more collagen fibers than the control groups [44].

Studies on larger animals, such as sheep, have also been performed to illustrate the effect of collagen on wound healing. For example, Melotti et al. manufactured collagen-based skin-like scaffolds (CBSSs) from collagen isolated from sea urchin food waste to treat skin wounds in sheep [69]. They showed that wounds treated with CBSS healed at a much faster rate than control groups. Beginning at day 14 post-injury, the treated groups displayed a significantly greater amount of keratinocyte migration compared to the untreated control groups. Furthermore, CBSS-treated groups had less inflammation over time and more deposition of granular tissue than the control group [69]. Gene expression analysis showed an upregulation of an important growth factor, VEGF-A, in the treated groups compared to the control [69]. In addition, hKER, a marker for hair follicle expression, was first observed at day 14 post-injury, only in the treated groups [69]. Taken together, these results indicate that collagen is a promising biomaterial for wound healing and skin regeneration.

**Figure 4:**
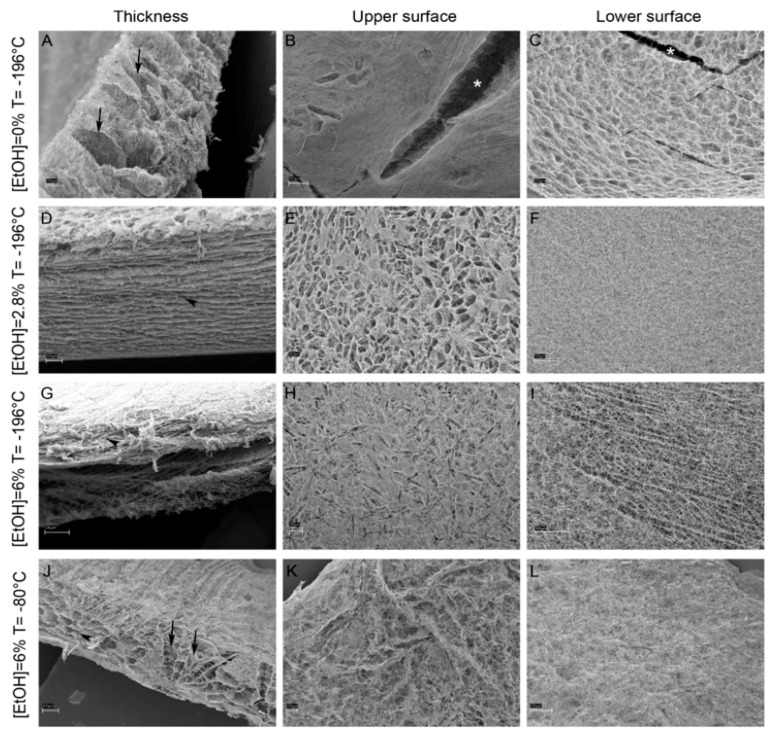
The 3D thickness and inner collagen structure of CBSS are shown in 0%, 2.8%, and 6% ethanol concentrations at different freezing temperatures (−80 °C and −196 °C). Scaffolds produced with 6% ethanol at −80 °C showed the highest mechanical stability compared to scaffolds produced with lower ethanol concentrations. First column: 3D scaffold thickness. Second column: 3D scaffold upper surface. Third column: 3D scaffold lower surface. Arrows: vertical channels; arrowheads: horizontal laminae; * scaffold macroscopic ruptures. Scale bars: (**A**,**D**,**F**,**I**) = 100 µm; (**B**,**C**,**E**,**G**,**H**,**K**,**L**) = 200 µm; (**J**) = 300 µm [21].

In another experiment by Ferrario et al., the integrity and performance of 3D-produced CBSS were evaluated under the condition of adding varying concentrations of ethanol and different freezing temperatures [21]. Their experimental results showed that the addition of ethanol in the process of producing collagen-based scaffolds at different temperatures can increase the thickness of these scaffolds, and the density of collagen fibers in the upper and lower surfaces of these scaffolds could also be enhanced (Figure 4). These factors ultimately led to greater mechanical stability and reduced deformation of this scaffold in cell culture and in vivo wound microenvironments [21].

**Figure 5:**
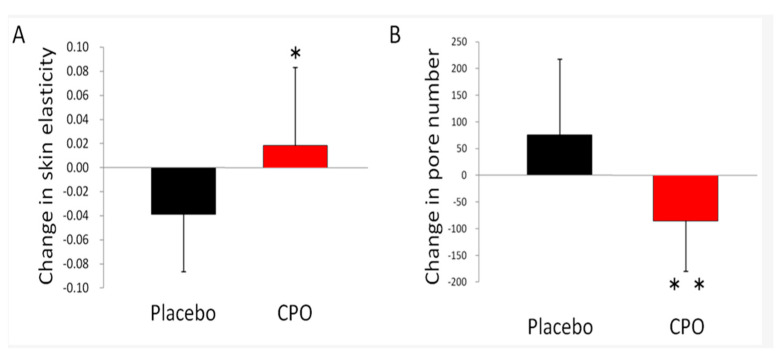
Image depicts the effect of dietary supplementation with fish-derived ornithine (CPO) on (**A**) skin elasticity and (**B**) pore number [45]. Placebo is shown as the black bars and the CPO group is shown as the red bars. Data are shown as the mean +/− SD. * Significant difference.

The biomedical applications of collagen for the purpose of skin regeneration are further prevalent in the anti-aging industry, as age-related changes associated with the skin can negatively impact aesthetic aspects of skin structure [70]. The elasticity, mechanical strength and overall structure of the skin are mainly maintained by collagen and elastin fibers [71,72]. Collagen synthesis decreases as one age, and this is one of the dominant contributions to changes associated with aging skin [73,74]. Many compounds, such as dietary vitamins, fatty acids, and essential amino acids, have been proven beneficial to skin health and appearance [75,76,77]. However, plant-derived dietary compounds are limited by their low bioavailability through the intestinal barrier and high metabolism rates [78]. Promisingly, marine collagen and collagen peptides have high bioavailability, potency, and a favorable safety profile [79,80]. A study performed by Ito et al. demonstrated that dietary supplementation with collagen peptide and ornithine (CPO) derived from fish significantly reduced trans-epidermal water loss and skin pore number and increased elasticity in the CPO-treated groups (Figure 5) [45].

Furthermore, aging is known to be associated with decreased growth hormone levels. Insulin-like growth factor-1 (IGF-1) levels are related to higher growth hormone secretion, and it was found that IGF-1 levels were significantly elevated from baseline in the CPO group [45]. The study also reported no adverse effects in response to one 30 mL CPO dietary supplement [45]. Similarly, De Luca et al. found that after taking 570 mg collagen peptides isolated from fish, participants had enhanced dermal thickness and acoustic density, an ultrasonic marker [46]. In addition, skin elasticity and sebum production were also significantly increased after collagen peptide supplementation, and no toxic effects were observed [45]. Alves et al. demonstrated that collagen type I isolated from salmon and codfish skins displayed no irritation on human skin [81]. The cytokines interleukin (IL)-6 and IL-8 were used as markers for inflammation and irritation in the study, and it was observed that there was no cytokine release after collagen administration [81]. In addition, collagen is high in the amino acids glycine, proline, and hydroxyproline, which contribute to the stability of collagen and its ability to withstand high temperatures due to a high number of hydrogen bonds [81]. Collagen was also able to retain a significant amount of water, which is important for its use in cosmetics [81].

Evans et al. conducted a randomized and triple-blinded clinical trial on 45–60-year-old women to demonstrate the impact of collagen from a freshwater fish on wrinkled skin and elasticity [82]. After three months of taking the collagen supplement, there was a significant reduction (35%) in wrinkles of participants compared to baseline [82]. It was also observed that cheek skin elasticity significantly improved at 6 weeks post-treatment. Furthermore, compared to the placebo group, it was noted that in addition to elasticity and wrinkle improvements, there was a 14% improvement in hydration, 22% improvement in radiance, and a 25% improvement in skin firmness [82]. In another experiment using chronologically aged mice, topical application or oral consumption of collagen hydrolysates was shown to have positive effects on skin aging [83]. Six months post-treatment, there were significant increases in collagen content and important antioxidant enzymes, including superoxide dismutase and glutathione peroxidase. Together, these factors improved the structure and appearance of the skin. It was found that when treated with collagen, the density and spread of type I and type III collagen fibers improved the structure of the epidermis and dermis [83]. Pei et al. also found that when aged mice were treated with marine collagen peptides, epidermal thickness, fibroblast activity, and antioxidant enzyme activity increased [84].

**Figure 6:**
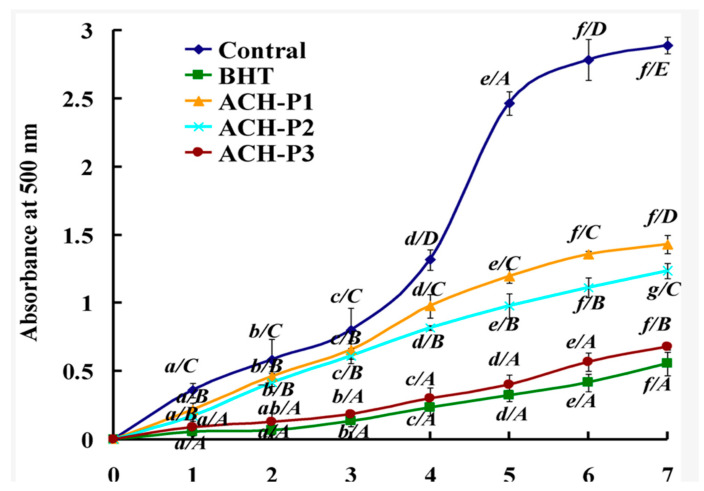
Inhibition of lipid peroxidation by three marine collagen peptides, ACH-P1, P2, and P3, compared to the known inhibitor BHT [89].

These antioxidant properties of marine collagen are promising as highly reactive and unstable free radicals, and oxidizing species damage cellular membranes, DNA, and macromolecules of skin cells, and are known to be heavily involved in skin aging [85,86,87]. Antioxidant enzymes such as superoxide dismutase and glutathione peroxidase are significant in that they protect against oxidative stress by inhibiting free radicals and other dangerous oxygen species [88]. Chi et al. isolated acid-soluble collagen from the scales of croceine croaker and demonstrated the antioxidant capabilities of three collagen peptides [89]. Upon using an oxygen radical absorbance capacity (ORAC), it was shown that collagen peptides displayed protective effects against hydroxyl, superoxide, and other radicals. Furthermore, the effect of collagen peptides (ACH-P1, P2, P3) on lipid peroxidation, a pathway involved in the formation of oxidant species, was investigated. As seen in Figure 6, compared to controls, lipid peroxidation was significantly reduced because of a decrease in absorbance, which measures the degree of oxidation [89]. Collagen peptides performed similarly to BHT, a known and effective antioxidant [89].

Taken together, it is evident that marine collagen is a successful and promising biomaterial for wound healing and cosmetics.

## 3. The Potential Role of Collagen in Bone and Cartilage Regeneration

Marine collagen sources serve not only as a promising avenue for healing skin injuries but also for bone-related trauma and regeneration. Bone fracture repair and healing is a form of tissue regeneration and is a complex process involving bone formation and breakdown [90,91]. Often, patients present with conditions that require reconstruction of large bones as a result of genetic abnormalities, trauma, infection, and tumors [92]. There is an increasing demand to improve methods of bone repair and regeneration, such as functional bone grafts [93].

Marine collagen bioactive peptides are known to aid in the absorption of calcium and zinc, which are important components of bone and are beneficial for osteoporosis prevention [94,95]. A study performed by Xu et al found that marine collagen peptides isolated and derived by hydrolysis from chum salmon increased serum osteocalcin in treated rats compared to controls. Osteocalcin is a protein hormone secreted by osteoblasts and plays a role in bone maintenance and regeneration through interaction with calcium. The study also found that bone organic matrix, density, femoral length, and femur mineral ions were significantly higher in the collagen-treated group than in the controls [94]. It was hypothesized that the increase in bone mineral density was likely due to increased osteoblast activity, as seen by the increase in bone size and serum osteocalcin [94]. These results shed light on the potential collagen peptides involved in mineral deposition, bone matrix development and an increase in osteoblastic activity, which strongly suggests that collagen is a promising biomaterial for the prevention and treatment of osteoporosis [94]. Osteoporosis and net bone loss are prevalent among aging women going through menopause resulting from estrogen deficiency [49]. Nomura et al. demonstrated that 20 mg of collagen isolated from shark gelatin also increased the bone mineral density of the spongy bone in rat models of menopausal osteoporosis [49].

**Figure 7:**
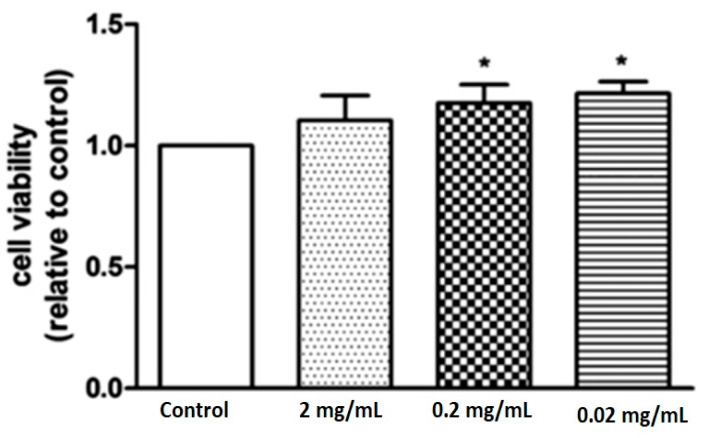
Figure shows the cell viability of rat-derived bone marrow mesenchymal stem cells in the control and marine collagen-treated groups [50]. The results show a significant increase in cell viability at 0.2 and 0.02 mg/mL in the collagen-treated groups (marked with *). The 2 mg/mL-treated group revealed no significant differences due to the high dose resulting in a complex negative feedback mechanism that suppressed cell proliferation.

Furthermore, the biological effect of marine collagen on rat-derived bone marrow stem cells has also been demonstrated. Liu et al. showed that 0.2 mg/mL collagen isolated from fish promoted cell survival and upregulated the expression of several osteogenic and endothelial markers [50]. As shown in Figure 7, there was a significant increase in cell viability at 0.2 and 0.02 mg/mL in the collagen-treated groups [50]. Interestingly, the 2 mg/mL-treated group showed no significant differences due to the high dose resulting in a complex negative feedback mechanism that suppressed cell proliferation [50].

In addition, osteogenic markers, such as alkaline phosphatase (which enhances the differentiation of cells into osteoblast/bone-forming cells), were significantly upregulated in the collagen-treated groups at 3 and 10 days post-treatment [50]. Similar to this study, Elango et al. found that collagen-treated bone marrow stem cells and mature osteoblastic cells depicted dose-dependent increased proliferation compared to controls [52]. Additionally, osteogenic marker mRNA and protein expression significantly increased in the treated groups compared to controls [52]. These results suggest that collagen is able to promote stem cell differentiation and osteoblastic activity. Yamada et al. also showed that marine collagen peptides extracted from both bone and skin of fish were able to increase osteoblastic cell proliferation, expression of osteogenic markers and mineral deposition [96].

In addition to the use of hydrolyzed collagen peptides, collagen scaffold structures have been shown to be beneficial with regards to bone regeneration. Diogo et al. found that collagen-calcium phosphate scaffold structures crosslinked with EDC/NHS supported the attachment and production of bone-building cells [97]. A more recent study found that within the jellyfish collagen scaffolds, there was greater de novo bone formation and increased macrophage recruitment compared to control groups [98]. Inflammatory cells, such as macrophages, are known to promote tissue repair, regulate inflammation and homeostasis, which is promising for bone tissue regeneration [98]. Similar to the above studies, Rachmawati et al. found that collagen scaffolds isolated from the *Aurelia aurita* jellyfish helped regenerate alveolar bone [99]. When treated with the collagen scaffold, there was increased osteoblasts and decreased osteoclasts compared to the control group suggesting a potential for alveolar bone regeneration. STRO-1, a biomarker for mesenchymal stem cells and osteocalcin, a protein hormone synthesized by osteoblasts were also increased in the collagen treated groups. These results suggest promising bone regeneration properties [99].

Marine sponges, also known as poriferans, serve as an important source of collagen and have a structure that resembles the cancellous architecture of bone tissue. Lin et al. conducted an in vitro assay using fibrinous collagen isolated from the *Callyspongiidae* marine sponge [100]. The study observed osteoblasts were able to anchor onto the surface of sponge fibers, proliferate, and grow on the cell-sponge constructs. The study also assessed the osteoconductive potential of the sponge collagen scaffold constructs and found that after 7 days the gene expression of two osteogenic markers, osteocalcin, and osteopontin, significantly increased [100]. On day 14, alkaline phosphatase gene expression, an indicator of osteoblastic differentiation, also significantly increased [100]. Similarly, Green et al. also utilized a marine sponge skeleton scaffold to assess whether collagen could induce osteogenesis [101]. The study found that human osteoprogenitor cells were able to attach to the scaffold within 16 h, and by 21 days, osteoprogenitor cells secreted an extracellular matrix. Furthermore, at 9 and 14 days, alkaline phosphatase activity significantly increased compared to controls [101]. All together, these results indicate that collagen fibers in the marine sponge skeleton provide a scaffold framework for osteoblast attachment, proliferation, and migration, which suggest a promising potential for use in bone tissue engineering [100,101].

The biomedical applications of marine collagen are not limited to skin and bone but also encompass cartilage regeneration. Osteoarthritis (OA) is characterized by a disturbance in cartilage homeostasis, which lacks self-repair and regenerative potential [102,103]. In OA, degradation of the cartilage occurs, which results in exposure of the subchondral bone, and this negatively impacts ones quality of life due to painful and stiff joints [103]. Promisingly, marine collagen has been shown to induce chondrogenic differentiation, paving the pathway for potential cartilage regeneration. Raabe et al. found that hydrolyzed fish collagen as well as the growth factor TGFB1 induced proteoglycan and collagen fiber synthesis [104]. Fish collagen also induced chondrogenic differentiation [104]. Similarly, Bourdon et al. investigated the effects of three collagen hydrolysates from fish skin and cartilage on the breakdown of chondrocytes [55]. The study found that 0.5, 50 and 100 µg/mL collagen hydrolysates elevated the level of collagen type I and II collagen. In addition, collagen-treated cells had decreased expression of protease markers known to be involved in OA development, Htra1, Mmp103, Adamts5 and Cox2 [55]. Ohnishi et al. also found that rabbits administered a combination of fish collagen peptides and glucosamine were protected from induced cartilage degradation (OA), whereas control groups developed OA [105]. It was found that fish collagen peptide and glucosamine, which are present in large amounts in connective tissue, and help maintain cartilage structure and integrity, had some protective effects alone, but their combined effects provided the most protection against OA [105].

**Figure 8:**
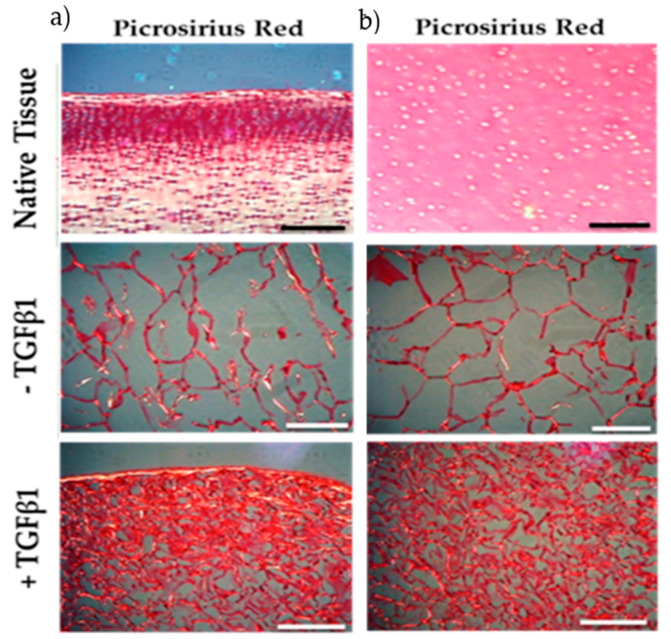
Histologic results show the amount and orientation of collagen fibers in native bovine tissue (containing immature chondrocytes) and chondrogenic tissue derived from bovine cartilage in the presence and absence of TGFβ1. Tissues are seeded on jellyfish collagen scaffolds. Collagen fibers were identified by picrosirius red staining. (**a**) Surface (**b**) center of scaffolds. Scale bars: 0.1 mm [106].

In a histological experiment by Ahmed et al., the effect of collagen from jellyfish sponge scaffolds on the chondrogenicity of bovine cartilage was observed [106]. Chondrogenicity is a complex process involving the proliferation and differentiation of chondroprogenitors and deposition of the extracellular matrix (ECM) [106]. In this experiment, chondrocytes derived from bovine cartilage tissue were seeded on jellyfish scaffolds to evaluate the amount of collagen deposition, and picrosirius red dye was applied to observe the content and orientation of collagen fibers (Figure 8) [106]. They used three different culture media: native tissue (bovine-derived immature cartilage) and bovine cartilage tissue containing chondroprogenitor cells in the presence and absence of transforming growth factor-β1 (TGFβ1) [106]. TGFβ1 has been shown to be an effective growth factor in cartilage formation and is present at high levels in healthy cartilage, but its level is greatly reduced in the cartilage of OA patients [106]. Staining results showed that in native bovine tissue, collagen fibers are mainly located on the tissue surface, but in chondrogenic culture, both at the tissue surface and in deeper areas, deposition of collagen fibers is more visible. Moreover, the addition of TGFβ1 to the culture medium further contributed to increasing the deposition of collagen fibers [106]. Taken together, the present data support the application of marine collagen in cartilage regeneration.

## 4. Advantages and Limitations Associated with Marine Collagen Use

Marine resources of collagen have many advantages over land animals and other sources. Not only are they available in abundance, have no religious constraints and are easily accessible, there have been few reported toxic effects at effective doses [32,33]. This is significant as a major source of collagen is from cattle, which have a risk of transmitting highly dangerous BSE and TSE [29,35]. In addition to its promising safety profile, the use of marine collagen is environmentally friendly. Fish skin, bones, and scales are vast sources of collagen, yet they are often discarded by seafood processing industries [30]. By using marine collagen, useful waste is reduced, and no further organisms are harmed in the isolation of collagen. Furthermore, collagen has a variety of applications in many fields, such as drug delivery, wound healing, skin aging, and tissue regeneration. Marine collagen was also shown to be as effective as sham collagen. In the example mentioned above, marine collagen was as effective as the currently administered antioxidant BHT [89]. In addition, other comparisons of sponge collagen membrane versus polyurethane membrane on healing of grant donor sites depicted that collagen use significantly increased wound healing quality and reduced healing time [107]. Marine collagens are also hydrolyzed more easily than mammalian collagen, which makes them more suited for further processing into peptide derivatives [108]. Furthermore, collagen has both structural and functional properties that make it a natural substrate for cell attachment, growth, and differentiation [109]. It is important to note, however, that although minor, some limitations do exist. It has been shown that marine collagen is less thermally stable than collagen from bovines, as they have fewer proline and hydroxyproline residues [110]. Additionally, most studies have investigated marine collagen’s efficacy in vitro or in animal models; however, more studies are needed that investigate the efficacy and potential adverse effects of marine collagen on human skin. Overall, the few limitations of marine collagen are strongly outweighed by their wide variety of benefits.

## 5. Concluding Remarks

The present review highlights the biomedical applications of marine collagen in wound healing, skin antiaging, and bone and cartilage regeneration. It is evident that marine collagen sources are significantly more advantageous than land animal sources. The ability of marine collagen to promote skin re-epithelization, vascularization, fibroblast migration, and overall faster wound healing rates has been demonstrated. Furthermore, the antiaging effects of marine collagen related to greater skin elasticity and wrinkle reduction are highly promising for the cosmetic industry. Furthermore, the significant impact on osteoporosis prevention and treatment by increasing bone density and mineral deposition is also evident. Bone-related diseases such as osteoporosis and OA can negatively impact one’s quality of life. Marine collagen and its derivatives were shown to delay and protect against OA and, thus, reduce mortality outcomes. Therefore, there should be continued investigation and discoveries of marine collagen sources, as they have thus far proven extremely beneficial.
